# Vaginal metastasis of pancreatic cancer

**DOI:** 10.11604/pamj.2015.20.124.5927

**Published:** 2015-02-12

**Authors:** Khadija Benhayoune, Hinde El Fatemi, Meryem El Ghaouti, Abdelaziz Bannani, Abdelilah Melhouf, Taoufik Harmouch

**Affiliations:** 1 Department of Surgical Pathology, CHU Hassan II, Fez, Morocco; 2 Departement of Gyneacology I, CHU Hassan II, Fez, Morocco; 3 Departement of Gyneacology II, CHU Hassan II, Fez, Morocco y

**Keywords:** Vagin, pancreatic cancer, metastases

## Abstract

Vaginal metastasis from pancreatic cancer is an extreme case and often indicates a poor prognosis. We present a case of pancreatic carcinoma with metastasis to the vagina that was discovered by vaginal bleeding. To our knowledge, this is the third case in the world of a primary pancreatic adenocarcinoma discovered of symptoms from a vaginal metastasis.

## Introduction

Pancreatic cancer is among the most devasting of humain malignancies. One of the major hallmarks of pancreatic cancer is its early systemic dessimination and its extraordinary local tumor progression. The usual sites of metastases in pancreatic cancer are the liver and peritoneal cavity. Other less common sites are the lung, bone and brain. Unusual sites such as muscle, skin, heart, pleura, stomach, umblicus, kidney, appendix, spermatic cord and prostate have also been reported [1–4] To our knowledge, the formation of metastases in the vagina from primary pancreatic cancer is exceptional. We report the third case in the world of a primary pancreatic adenocarcinoma discovered of symptoms from a vaginal metastasis [5, 6]

## Patient and observation

**Figure 1:**
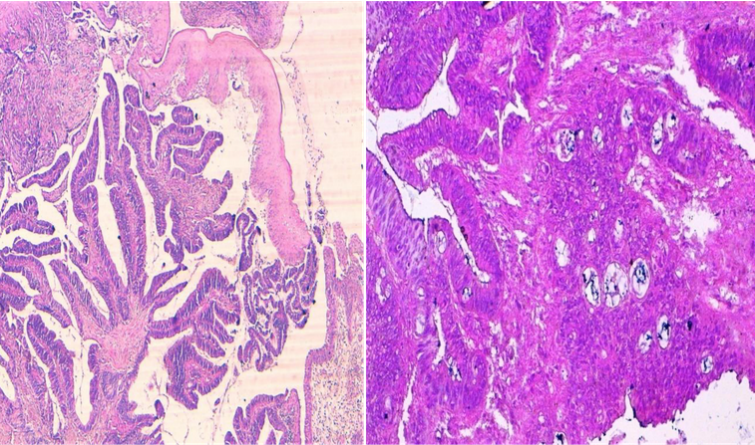
Well-differentiated, mucinous adenocarcinoma (HES ×4 and HES x 20)

**Figure 2:**
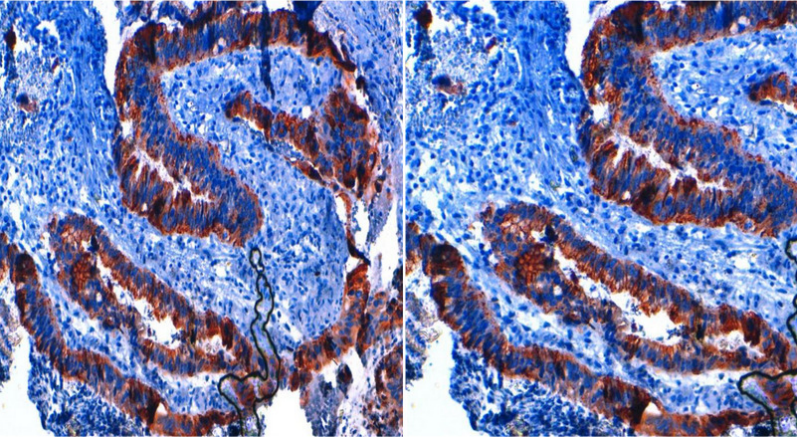
CK19 and CK20 immunohistochemestry highlighting tumor cells in the well differentiated pancreas tumor

**Figure 3:**
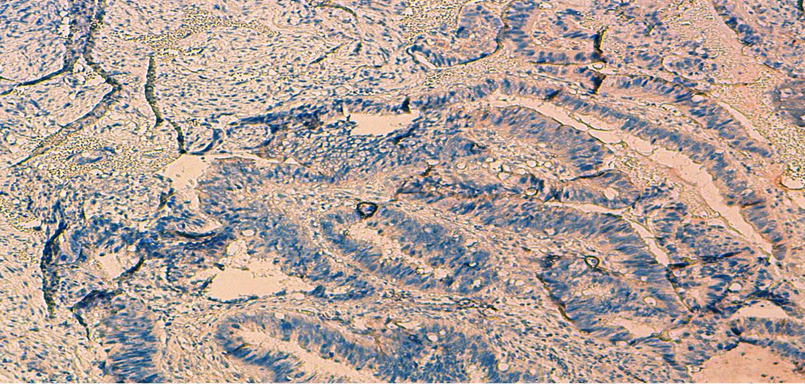
The tumor cells are negative for CK7

**Figure 4:**
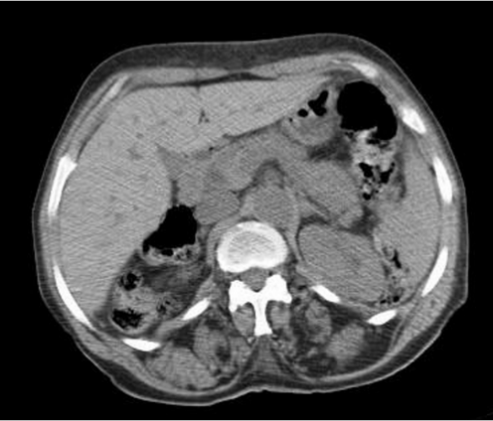
Metastatic tumor presenting as possible primary lesions, extragenital and genital primaries

70-year-old woman presented to Department of Gynecology with vaginal bleeding without other associated signs. Physical examination revealed a friable mass in the anterior wall of vagina and invading the clitoris. Bimanual pelvic examination showed that cervix, uterus and bilateral adnexal structures are normal. Speculum examination showed an ulcerative and exophytic mass, 6cm in diameter on the anterior wall of vagina at third lower. The lymph nodes are free. Transabdominal pelvic ultrasound reported that the uterus was with homogenous myometrium, indistinct endometrial echo pattern and bilateral adnexal areas were normal. Incisional biopsy was performed and reported a well-differentiated, mucinous adenocarcinoma with a complex gland pattern with areas of cribriform architecture, stratified and hyperchromatic nuclei, and moderate nuclear atypia (Figure 1). Tumor cells are immunoreactive for CK20 and CK19. They are negative for CK7, CK5/6, CK14, CD10 and hormone receptors (Figure 2, Figure 3). At the same time, an abdominopelvic CT was performed. It showed the presence of a tumor mass in the pancreatic tail (Figure 4). Response to radiological and histological findings, the diagnosis of vaginal metastasis of pancreatic cancer has been retained.

## Discussion

Extragenital tumor metastatic to the female genital tract is a rare occurrence. Few cases are reported in literature describing isolated metastasis to the vagina. To our knowledge, this is the first case of a reveal vaginal metastasis of pancreatic cancer. One report analysed the origin of metastases in the female genital tract; in 149 out of 325 cases, metastases originated from extra- or intragenital primaries [7, 8] and they were more frequently localized in the ovaries [9, 10] and vagina (Table 1). The majority of the extragenital metastases were adenocarcinomas from the gastrointestinal system, but a variety of other primaries did occasionally spread to the genital tract. Cancer metastases to the vagina from upper abdominal organs are quite rare, especially in the absence of metastatic disease to other abdominal or pelvic structures. Vaginal metastases can be difficult to detect, depending on their size and location in the vagina. So, the histological examination is essential for the positive diagnosis. Cells tumor of metastatic pancreatic adenocarcinoma show positive immunohistochemical staining for SMAD4, CK19, CK7, CK18, CK20 and CA19/9. It is diffuclt to conceptualize how a pancreatic malignancy could manifest clinically as a vaginal metastasis without evidence of other organ metastasis or significant abdominal symptoms. Metastatic tumors may appear in locations that are not in the line of direct spread from their primary focus, which is called paradoxical metastasis. When direct contiguous spread is not the case, it is speculated that remote vaginal metastasis may occur either through lymphatic or hematogenous routes. The valveless “vertebral plexus of Baston”communicates above the intracranial venous sinuses and segmentally with the veins of the thorax, abdomen and pelvis. This vertebral venous system has been well documented as a pathway of paradoxical metastasis, as in bone metastases in patients with prostate cancer and breast cancer, which were also described by Baston [11]. Because this plexus allows for tumor cells to spread in both on anterograde and retrograde fashion, we theorize that the pancreatic tumor metastasized to the vagina via this hematogenous route. Disseminated metastatic disease is frequently present in patients with vaginal metastases and the prognosis is extremely poor in these patients. Our case is exceptional. Indeed, vaginal localization was indicative of primary pancreatic tumor, without other sites. A distinction from primary vaginal adenocarcinoma of the intestinal type may be difficult. Extensive research should be performed to exclude a primary adenocarcinoma of another location because of differences prognosis and treatment. A broad immunohistochemical panel should assist in making the correct diagnosis. Although it is frequently associated with primary vaginal tumors, vaginal bleeding or discharge might be the first clinical manifestations of an occult carcinoma or clinical signs of a wide spread metastatic disease. Data on the treatment of vaginal metastasis are limited but most cases are treated with radiotherapy [12]. In the absence of any other metastatic lesion, treatment of both the primary tumor as well as the isolated metastasis should be carried out with a surgical approach and consideration for curative radiotherapy [13].

**Table 1:** Metastatic tumor presenting as possible primary lesions, extragenital and genital primaries

Primary sites	Site of metastasis					
	ovary	salpinx	endometrium	cervix	vagina	vulva
Colon, rectum	18	0	1	1	7	0
Appendix	1	0	1	0	1	0
Stomach	4	0	1	1	0	0
Breast	3	0	2	0	3	1
Uncertain	8^1^	0	1	0	1	1
Miscellanous	6^2^	0	0	2^3^	2^4^	1^5^
Pancreas	-	-	-	-	3	-
Ovary	-	-	1	0	1	0
Salpinx	1	1	-	-	0	6
Endometrium	2	1	-	-	-	6
Cervix	0	0	0	-	-	-
Vagina	0	0	0	0	-	-
Vulva	43	2	10	5	16	16

## Conclusion

Metastases to the female genital tract from pancreatic cancer are unsual. The incidence of vagina metastases secondary to adenocarcinoma of the exocrine pancreas is unknown. Symptom producing vagina metastases are common than primary site in patients who present a metastatic adenocarcinoma of unknown origin.
